# Modulating the Plant Microbiome: Effects of Seed Inoculation with Endophytic Bacteria on Microbial Diversity and Growth Enhancement in Pea Plants

**DOI:** 10.3390/microorganisms13030570

**Published:** 2025-03-03

**Authors:** Shervin Hadian, Donald L. Smith, Skaidrė Supronienė

**Affiliations:** 1 Microbiology Laboratory, Institute of Agriculture, Lithuanian Research Centre for Agriculture and Forestry, Instituto Ave. 1, Akademija, LT-58344 Kėdainiai, Lithuania; skaidre.suproniene@lammc.lt; 2 Department of Plant Science, McGill University, Montreal, QC H9X 3V9, Canada

**Keywords:** *Bacillus*, microbial diversity, metagenomic analysis, pea

## Abstract

Understanding plant microbe interactions is crucial for achieving sustainable agriculture. This study investigated the effects of inoculating pea plants (*Pisum sativum*) with two endophytic *Bacillus* strains, AR11 and AR32, isolated from *Artemisia* species and characterized by phosphate solubilization, nitrogen fixation, and pathogen antagonism. Utilizing cutting-edge methods such as rarefaction curves, rank abundance modeling, and metagenomic analysis, this research provides a detailed understanding of how these bacterial strains influence plant associated microbiomes. AR11 significantly enhanced microbial diversity, while AR32 showed a moderate effect. Beta diversity analyses revealed distinct shifts in microbial community composition, with AR11-treated samples enriched with beneficial taxa such as *Paenibacillus*, *Flavobacterium*, and *Methylotenera*, known for their roles in nutrient cycling, pathogen suppression, and plant health promotion. This innovative methodological framework surpasses traditional approaches by offering a comprehensive view of ecological and functional microbiome shifts. The study highlights the potential of nonhost bacteria as biostimulants and their role in developing microbiome engineering strategies to enhance plant resilience. These findings contribute to sustainable agriculture by demonstrating how microbial inoculants can be employed to enhance crop productivity and environmental resilience in diverse agricultural systems.

## 1. Introduction

Endophytic bacteria are important microorganisms that live within plant tissues and play a key role in promoting plant growth, health, and nutrient uptake. These bacteria are found in all plant species, forming beneficial relationships with their hosts. They integrate into the plant’s metabolic processes and produce signaling molecules that enhance plant functions. Some endophytes can also produce similar compounds to their host plants, even when grown separately, highlighting their potential for agricultural and medicinal applications [1,2,3]. Endophytic bacteria promote plant growth through both direct and indirect mechanisms. Directly, they improve nutrient absorption, produce phytohormones that regulate plant growth, and help plants tolerate environmental stress. Indirectly, they protect plants through static and cidal activities against harmful pathogens, producing antibiotics, and activating plant defense systems [4,5].

Medicinal plants, such as *Artemisia* spp., are a rich source of beneficial endophytes with diverse functional traits. Endophytes isolated from *Artemisia* spp. have demonstrated various beneficial properties, including phosphate solubilization, nitrogen fixation, and plant pathogen antagonism, making them valuable for sustainable agriculture. Extracts from *Artemisia absinthium* and endophytic bacteria associated with *Artemisia* spp. have been shown to function as effective biostimulants, enhancing plant productivity under diverse environmental conditions [6,7,8]. These findings highlight the significant role of *Artemisia* spp. in promoting crop resilience and productivity through their endophytic microbiota and bioactive compounds.

Plants and their microbiomes exhibit dynamic responses to anthropogenic environmental changes, adapting ecologically through shifts in microbial community composition and evolutionarily by modifying natural selection pressures. These interactions can drive reciprocal ecological and evolutionary feedbacks, particularly in environments where plant microbe relationships are functionally significant. Understanding these dynamics is crucial for advancing sustainable agriculture and conservation strategies [9].

Bacterial inoculation is increasingly recognized as an effective approach to enhancing plant-associated microbial diversity and advancing sustainable agricultural practices. By introducing specific endophytic bacterial strains, it is possible to modulate the composition and functionality of plant microbiomes, thereby improving plant health, productivity, and resilience to environmental challenges. Beneficial bacteria are widely studied for their capacity to enhance nutrient cycling, suppress pathogens, promote plant growth, and improve stress resilience. However, the mechanisms by which bacterial inoculation influences the assembly and composition of root and shoot endophytic microbiomes remain poorly understood. This study focused on seed inoculation with two *Bacillus* strains isolated from *Artemisia* species, selected for their demonstrated traits, such as phosphate solubilization, nitrogen fixation, and antagonistic activity against plant pathogens [6]. While metagenomic studies on plant microbe interactions have provided valuable insights, few have investigated the use of non-host *Bacillus* strains isolated from medicinal plants, such as *Artemisia* species, to enhance microbial diversity and promote plant resilience. Most existing research focuses on well-studied host-associated bacteria, leaving a gap in understanding the ecological and functional potential of non-host strains in agricultural applications.

The aim of this study was to investigate how inoculating pea plants with potentially endophytic *Bacillus* strains influences microbial diversity and fosters beneficial interactions within plant tissues. By uncovering the ecological and functional roles of these strains using advanced metagenomic methods, this research introduces a novel framework for harnessing non-host bacteria as biostimulants in sustainable agriculture and microbiome engineering.

## 2. Materials and Methods

### 2.1. Isolation and Selection of Endophytic Bacteria

The endophytic bacterial strains used in this study were isolated from four *Artemisia* species (*A. absinthium*, *A. campestris*, *A. dubia*, and *A. vulgaris*) collected from three distinct locations in Lithuania (Kaunas, Kėdainiai, and Šiauliai). Plant samples, collected during the leaf development stage, were surface-sterilized before microbial isolation. To confirm the effectiveness of surface sterilization, water from the final rinse step was plated on tryptic soy agar (TSA) medium (Sigma-Aldrich (Merck Group), St. Louis, MO, USA), and no bacterial growth was observed, verifying that the isolates were endophytic. Root, stem, and leaf tissues were sectioned and cultured on tryptic soy agar (TSA) plates to obtain bacterial isolates. Pure bacterial isolates were identified through 16S rDNA sequencing and characterized for plant growth-promoting traits such as phosphate solubilization, nitrogen fixation, and antagonistic activity against phytopathogens. From a total of sixty-two characterized isolates, two strains, AR11 and AR32, were selected for this study due to their strong phosphate solubilization, nitrogen fixation, and antagonistic activities against *Fusarium* spp. were previously evaluated and published. Both strains were originally isolated from the roots of *Artemisia absinthium* [6].

### 2.2. Seed Inoculation with Bacterial Strains

Pea seeds (*Pisum sativum* L.) were surface-sterilized using the standard protocol. First, seeds were immersing them in 70% ethanol for 1 min, to remove any external contaminants. Followed by 2.5% sodium hypochlorite for 10 min to eliminate surface microorganisms. Then, they were thoroughly rinsed five times with sterile distilled water to remove any residual ethanol and sodium hypochlorite. Bacterial suspensions of AR11 and AR32 were prepared for an optical density (OD600) of 1.0, corresponding to approximately 10^8^ CFU mL^−1^. Surface-sterilized seeds were immersed in the bacterial suspensions and incubated at room temperature for 3 h with gentle shaking to ensure uniform coating. Control seeds were treated with sterile distilled water using the same procedure. Treated and controlled seeds were sown in sterilized pots containing an autoclaved soil and maintained under controlled greenhouse conditions (25 ± 2 °C, 16 h photoperiod, light intensity of ~250 µmol m⁻^2^ s⁻^1^, 60% relative humidity). Three replications were considered for each treatment.

### 2.3. Soil Chemical Analysis

Prior to sowing, the sterilized soil was analyzed for chemical properties to ensure uniformity across treatments. Soil chemical analyses, including the determination of organic carbon (using the oxidation spectrophotometric method), total nitrogen (using the Kjeldahl method), pH (using the potentiometric method), and mobile phosphorus and potassium (using the ammonium lactate (A–L) method), were conducted at the LAMMC Chemical Research Laboratory.

### 2.4. DNA Extraction from Pea Plants

After 20 days of growth, during the leaf development phase (BBCH stages 11–19), pea plants from bacterium-inoculated groups (AR11: inoculated with bacterial strain AR11, AR32: inoculated with bacterial strain AR32) and the control group (C: without bacterial inoculation) were harvested. This time point was chosen as it marks the early vegetative stage when microbial colonization stabilizes, enabling the reliable assessment of endophytic communities [10,11]. Roots (R) and shoots (S) were separated and surface-sterilized by immersion in 70% ethanol for 2 min, followed by 5% sodium hypochlorite for 4 min, and thoroughly rinsed with sterile distilled water. Replicates (1, 2, 3) were processed for each group. Total DNA was extracted from sterilized tissues separately using the Quick DNA Plant/Seed Miniprep Kit (Zymo Research, Irvine, CA, USA) according to the manufacturer’s instructions. The extracted DNA was quantified and checked for purity using a ND-1000 Spectrophotometer (Nano Drop Technologies, Wilmington, NC, USA) and subsequently prepared for sequencing to assess the microbial diversity in plant tissues.

### 2.5. Microbial Community Analysis

#### 2.5.1. High-Throughput Sequencing

DNA samples were sent to Biomarker Technologies (BMK) company in Germany for high throughput sequencing using PacBio technology. The 16S rRNA region was amplified with primers 27F(AGRGTTTGATYNTGGCTCAG) and 1492R (TASGGHTACCTTGTTASGACTT). Quality filtering, primer trimming, sequence assembly, and chimera removal were performed using Trimmomatic (v0.33) [12], Cutadapt (v1.9.1) [13], USEARCH (v10) [14], and UCHIME (v8.1) [15], respectively. Sequences were filtered to retain high quality reads (16S rRNA: 1200–1650 bp).

#### 2.5.2. Alpha Diversity Analysis

Alpha diversity metrics, including Abundance-Based Coverage Estimator (ACE), Chao1, Shannon, Simpson, and PD Whole Tree indices, were calculated to assess microbial richness and evenness. Statistical analyses, including the Kruskal–Wallis test and Wilcoxon rank sum test, were performed to identify significant differences among AR11, AR32, and control groups. Diversity curves (rarefaction, rank abundance, and Shannon diversity) were generated using R packages (vegan v2.6-4, phyloseq v1.42.0, ggplot2 v3.4.0).

#### 2.5.3. Beta Diversity Analysis

Beta diversity was analyzed using Bray–Curtis, Binary Jaccard, Weighted UniFrac, and Unweighted UniFrac distance metrics. Ordination methods such as (Non-Metric Multidimensional Scaling (NMDS), Principal Coordinate Analysis (PCoA), and Principal Component Analysis (PCA) were used to visualize microbial community differences across treatments. Statistical significance of observed differences in microbial composition was determined using PERMANOVA and ANOSIM, with R^2^ values and *p*-values calculated using the adonis function from the vegan package (v2.6-4) in R program (v4.2.3).

#### 2.5.4. Taxonomic Annotation

Operational Taxonomic Units (OTUs) were clustered using a 97% similarity threshold and annotated using the SILVA database (Release 138, https://www.arb-silva.de/, accessed on 10 January 2024). Taxonomic annotations at the genus and phylum levels were visualized through bar charts, heatmaps, and phylogenetic trees. Ternary plots were created using the ggtern package (v3.3.5) in R to illustrate shifts in bacterial phyla across AR11, AR32, and control groups.

### 2.6. Microbial Functional Profiling

Metagenomic data were analyzed using predictive bioinformatics tools to infer microbial functional traits, including aerobic/anaerobic metabolism, mobile element presence, biofilm formation, and stress tolerance. Relative abundances of these functional groups were calculated for AR11, AR32, and control groups. Functional profiles were visualized using the ggplot2 package (v3.4.0) in R to compare the impact of bacterial inoculation on microbial community functions.

### 2.7. Microbial Community Network Analysis

Microbial co-occurrence networks were constructed using DADA2 processed 16S rRNA data. Spearman correlation coefficients (*p* < 0.05, |r| > 0.6) were used to identify significant relationships among bacterial genera. Networks were visualized using the R igraph (v1.3.5) and ggClusterNet packages (v1.0.1). Node sizes represented relative abundances, while edges denoted positive or negative correlations, with nodes color coded by phylum.

### 2.8. Statistical Analysis

All statistical analyses were conducted in R (v4.2.3, https://cran.r-project.org/, accessed on 10 January 2024). Statistical tests, including Kruskal–Wallis, Wilcoxon rank-sum, PERMANOVA, and ANOSIM, were performed using R packages vegan (v2.6-4), phyloseq (v1.42.0), and stats (base R v4.2.3). Visualizations, including bar charts, heatmaps, diversity curves, and network graphs, were generated using ggplot2 (v3.4.0), ggpubr (v0.6.0), ggtern (v3.3.5), and igraph (v1.3.5). Alpha and beta diversity indices, as well as functional and taxonomic analyses, were compared between AR11 (root and shoots), AR32 (root and shoots), and control groups to determine significant differences in microbial community composition and function.

### 2.9. Data Availability

The raw sequence data generated in this study have been deposited in the NCBI GenBank Sequence Read Archive (SRA) under Bio Project ID PRJNA1221229. The sequence data can be accessed publicly for further analysis.

## 3. Results

### 3.1. Soil Chemical Properties

The analysis of the soil chemical composition revealed some important parameters that gave insight into its fertility and suitability for vegetative growth and microbial interaction. The soil used in this study was amended with commercial compost, which explains the high percentage of organic carbon (11.99%) compared to natural soils, which typically contain only 1.5–3% organic carbon. Also, a remarkable level of nitrogen (1.084%), which both indicate a high content of organic matter in the soil. The soil pH was slightly alkaline (7.79), which is near ideal for many plant and microbial species, as this improves nutrient availability and microbial activities. Additionally, this soil also contained a significant number of essential macronutrients, with 460 mg kg^−1^ and 205 mg kg^−1^ concentrations for potassium oxide (K_2_O) and phosphorus pentoxide (P_2_O_5_), respectively. Such high amounts of potassium and phosphorus show the capability of this soil to allow for dynamic growth among plants and microbe interactions.

### 3.2. Impact of Early Inoculation on Microbial Community and Composition

#### 3.2.1. Sequencing Quality and OTU Diversity

- Shared and Unique Microbial Communities Across Treatments

**Figure 1:**
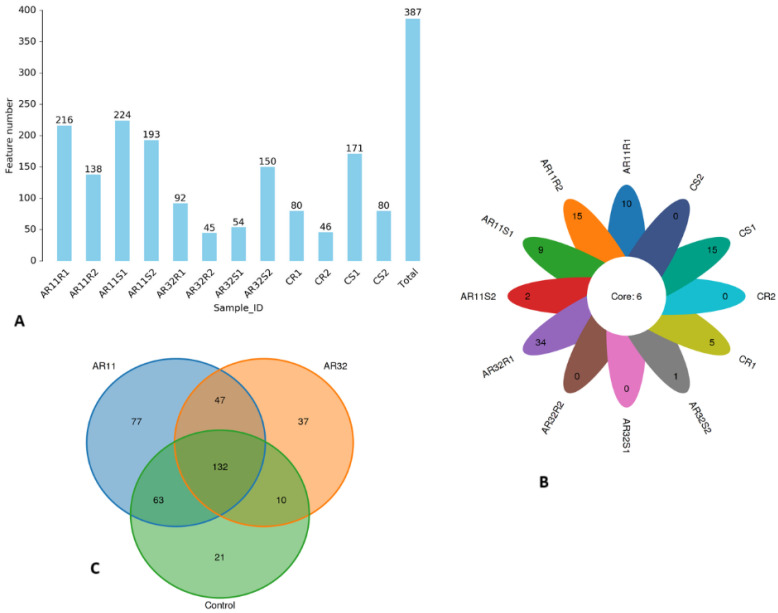
Microbial diversity across samples and treatments. (**A**) OTU bar chart: bar height represents the number of OTUs identified per sample. (**B**) Flower chart: each petal represents a unique OTU, with number inside the petal proportional to OTU count; the central circle indicates the core microbiome shared across all samples. (**C**) Venn diagram: shared and unique microbial species among AR11, AR32, and the control. AR11: samples inoculated with bacterial strain AR11, AR32: samples inoculated with bacterial strain AR32, control C: samples without bacterial inoculation. S: stem samples; R: root samples. 1, 2, 3: biological replicates.

We generated a total of 609,716 raw Circular Consensus Sequencing (CCS) reads across all samples. After applying strict quality filters, 609,680 high-quality reads were retained, and further processing refined these into 597,295 operational sequences with an average length of 1411 bp. The percentage of effective reads ranged from 90.2 to 99.9%, ensuring robust and reliable data for downstream analysis. Our OTU analysis revealed notable variations in microbial diversity among the samples, ranging from 45 to 224 OTUs (Figure 1A). The samples AR11S1 and AR11R1 had the highest diversity, with 224 and 216 OTUs, respectively, indicating a richer microbial community. In contrast, AR32R2 and CR2 showed the lowest diversity, with only 45 and 46 OTUs, respectively. These results highlight the significant impact of inoculation treatments and environmental conditions on microbial composition. To better illustrate these patterns, we created a flower chart where each petal represents a specific OTU. The numbers inside of the petals reflect the abundance of OTUs. Samples AR11S1 and AR11R1 showed the highest number in petals, underscoring their higher diversity, while AR32R2 and CR2 displayed lower numbers in petals, indicating lower diversity. At the center of the flower chart, the core microbiome is shown, representing microbial taxa shared across all samples (Figure 1B).

To gain deeper insight into the treatment level of microbial diversity, a Venn diagram was used to compare the distribution of shared and unique microbial species between AR11 and AR32 and the control groups. AR11 had the highest treatment specific diversity, with 77 species found only in this treatment group, while AR32 and the control group had 37 and 21 unique species, respectively. A core microbiome of 132 species was shared among all treatments, indicating a stable microbial community. Further analysis showed that AR11 shared 63 species with the control group, indicating a higher similarity in microbial composition than AR32, which shared only 10 species with the control. AR11 and AR32 shared 47 species independent of the control group. This accentuates the disparate impacts of AR11 and AR32 on microbial populations, with AR11 having more similarity with the control group, while AR32 promotes a more differentiated microbial composition, as visualized in Figure 1C.

### 3.3. Impact of Bacteria Inoculation (AR11 and AR32) on Bacterial Diversity and Richness in Pea Plants

#### 3.3.1. Alpha Diversity Results

**Figure 2:**
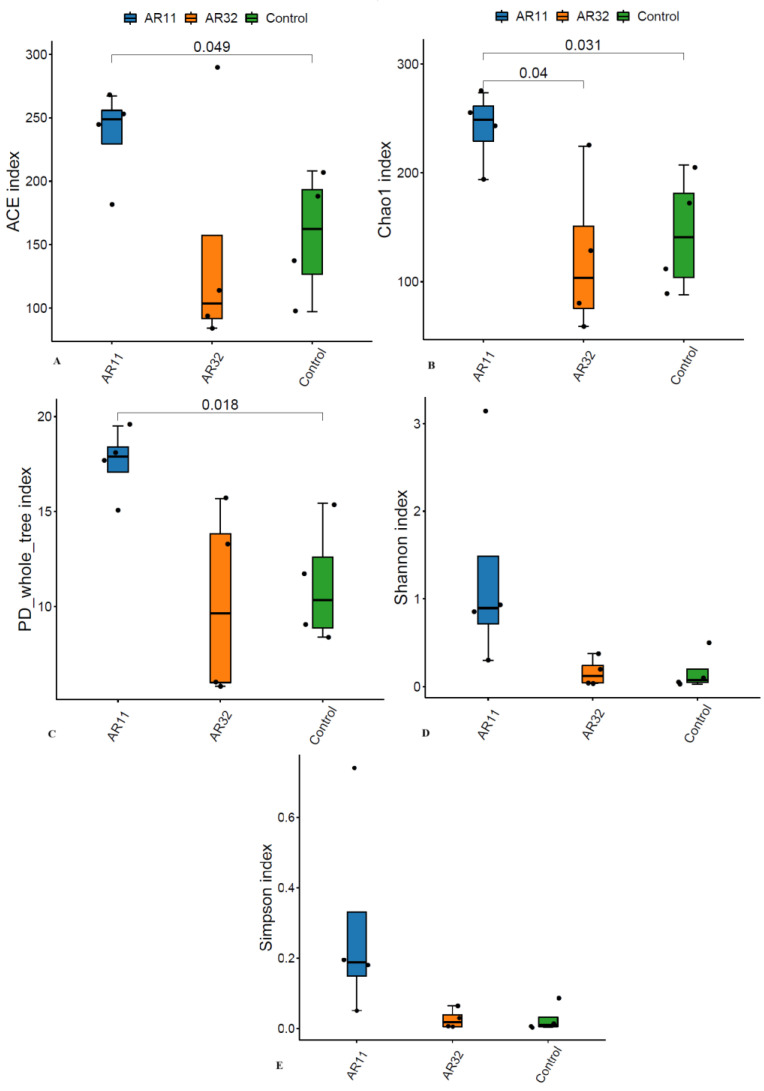
Impact of AR11 and AR32 treatments on alpha diversity indices, compared to the control group. (**A**) ACE index, representing species richness, (**B**) Chao1 index, another measure of species’ richness, (**C**) PD whole tree index, representing phylogenetic diversity, (**D**) Shannon index, reflecting richness and evenness, and (**E**) observed species, representing the total number of species observed. Statistical comparisons were performed using a two tailed *t*-test, with significant differences (*p* < 0.05). Error bars represent standard deviations. AR11 and AR32: inoculated plant with two different bacterial strains.

To evaluate the microbial richness and diversity of pea plants after *Bacillus* inoculation, several alpha diversity indices were calculated, including ACE, Chao1, Shannon, Simpson, and phylogenetic diversity (PD) whole tree. The ACE and Chao1 indices, which estimate species richness, showed significant differences (*p* < 0.05). AR11-treated plants had higher richness values than both the AR32-treated and control groups, indicating a greater number of bacterial species in AR11 inoculated samples (Figure 2A,B). The phylogenetic diversity, as measured by the PD whole tree index, was also significantly enhanced in AR11-treated plants compared to the AR32-treated and control groups (Figure 2C). While AR32 treatments increased phylogenetic diversity compared to the controls, the effect was less pronounced than with AR11. Similarly, the Shannon diversity index, which combines richness and evenness, indicated a more balanced microbial community under AR11 treatment (Figure 2D). In contrast, the Simpson index, which focuses more on evenness, showed no significant differences between treatments. However, the low Simpson index values across all groups suggested a balanced microbial community overall (Figure 2E).

#### 3.3.2. Impact of Inoculation on Microbial Richness and Community Structure

**Figure 3:**
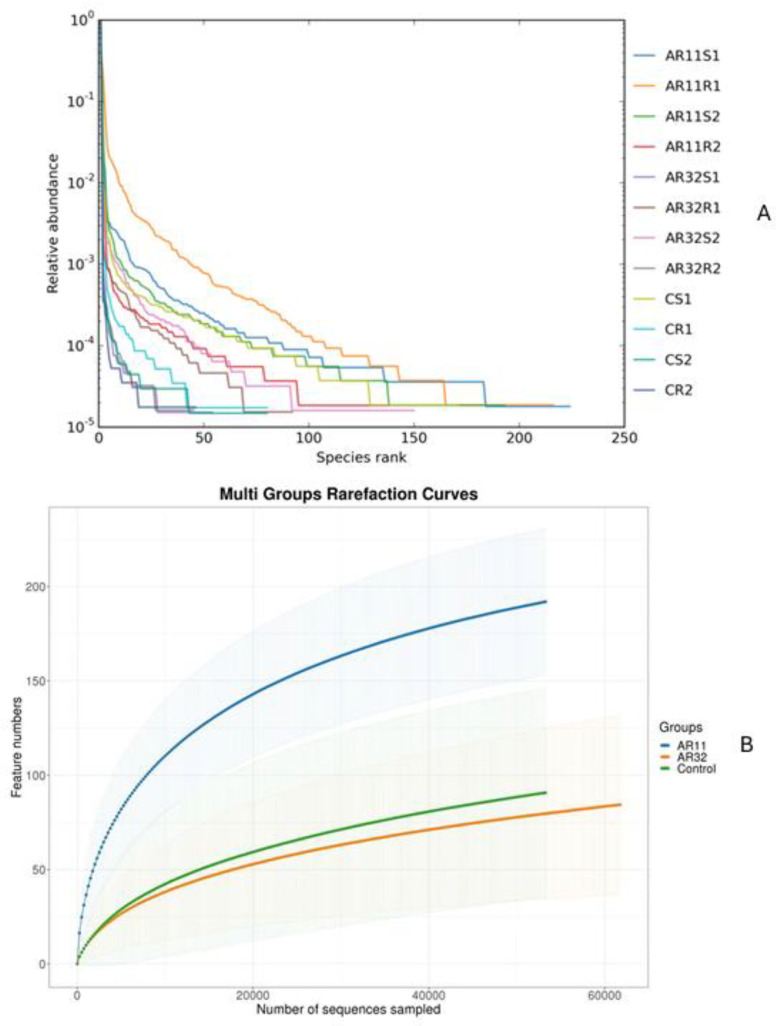
Effects of bacterial inoculation in enhancing microbial richness and diversity. (**A**) Rank abundance curves, (**B**) rarefaction curves, AR11 and AR32: inoculated plant with two different bacteria strains. AR11: samples inoculated with bacterial strain AR11, AR32: samples inoculated with bacterial strain AR32, control C: samples without bacterial inoculation. S: stem samples; R: root samples. 1, 2, 3: biological replicates.

Rarefaction curves showed that the sequencing depth was sufficient to capture the microbial diversity in all samples (Figure 3A). Samples treated with AR11 and AR32 reached plateau phases earlier, with AR11 showing the highest OTU counts, indicating greater microbial richness. While AR32 treatments also increased microbial diversity compared to the controls, the effects were less pronounced than those of AR11. Rank abundance curves further highlighted these differences in microbial community structure (Figure 3B).

AR11-treated samples exhibited longer and more gradual curves, reflecting greater species richness and evenness. In contrast, control samples had steeper curves, suggesting lower diversity and dominance by a few microbial taxa. Species accumulation curves confirmed that the sampling effort was sufficient and revealed trends in species richness. Samples treated with AR11 reached asymptotic levels more quickly, demonstrating better representation of the microbial community. The rapid leveling off of these curves in inoculated samples underscores the effectiveness of bacterial inoculation in boosting microbial richness and diversity. Overall, both AR11 and AR32 treatments significantly enhanced microbial richness, diversity, and evenness compared to the control groups, with AR11 showing the most substantial improvements.

#### 3.3.3. Beta Diversity Results

**Figure 4:**
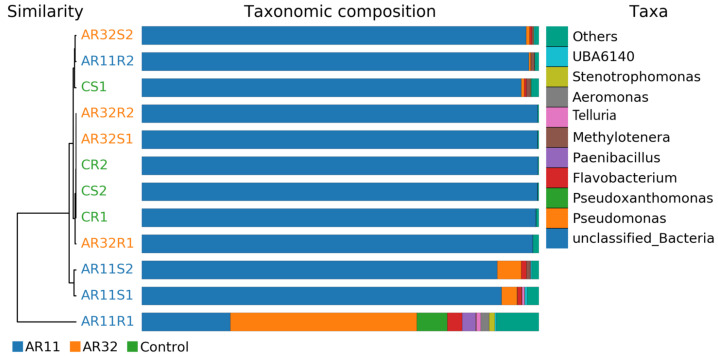
Hierarchical clustering is based on beta diversity measures (weighted UniFrac) to display relationships between microbial communities across treatments. AR11: samples inoculated with bacterial strain AR11, AR32: samples inoculated with bacterial strain AR32, control C: samples without bacterial inoculation. S: stem samples; R: root samples. 1, 2, 3: biological replicates.

Beta diversity analysis revealed significant differences in microbial community composition between the Control group and the groups inoculated with *Bacillus* strains AR11 and AR32. Multivariate analysis and clustering techniques highlighted inoculation-induced changes in community structure and function. Hierarchical clustering dendrograms and taxonomic composition bar charts (Figure 4) confirmed distinct clustering patterns, with AR11- and AR32-treated samples forming separate clusters from the Control, particularly evident in the weighted UniFrac metric, which integrates phylogenetic relationships and relative abundances. Supplementary Analyses (Figures S1 and S2) reinforced these findings, displaying consistent clustering across various metrics, including binary Jaccard, Bray–Curtis, unweighted UniFrac, and weighted UniFrac. The taxonomic composition analysis further revealed shifts in microbial taxa, such as increased relative abundance of *Methylotenera* in AR11-treated samples and *Paenibacillus* in AR32-treated samples, alongside variations in *Flavobacterium* and *Pseudomonas*.

**Figure 5:**
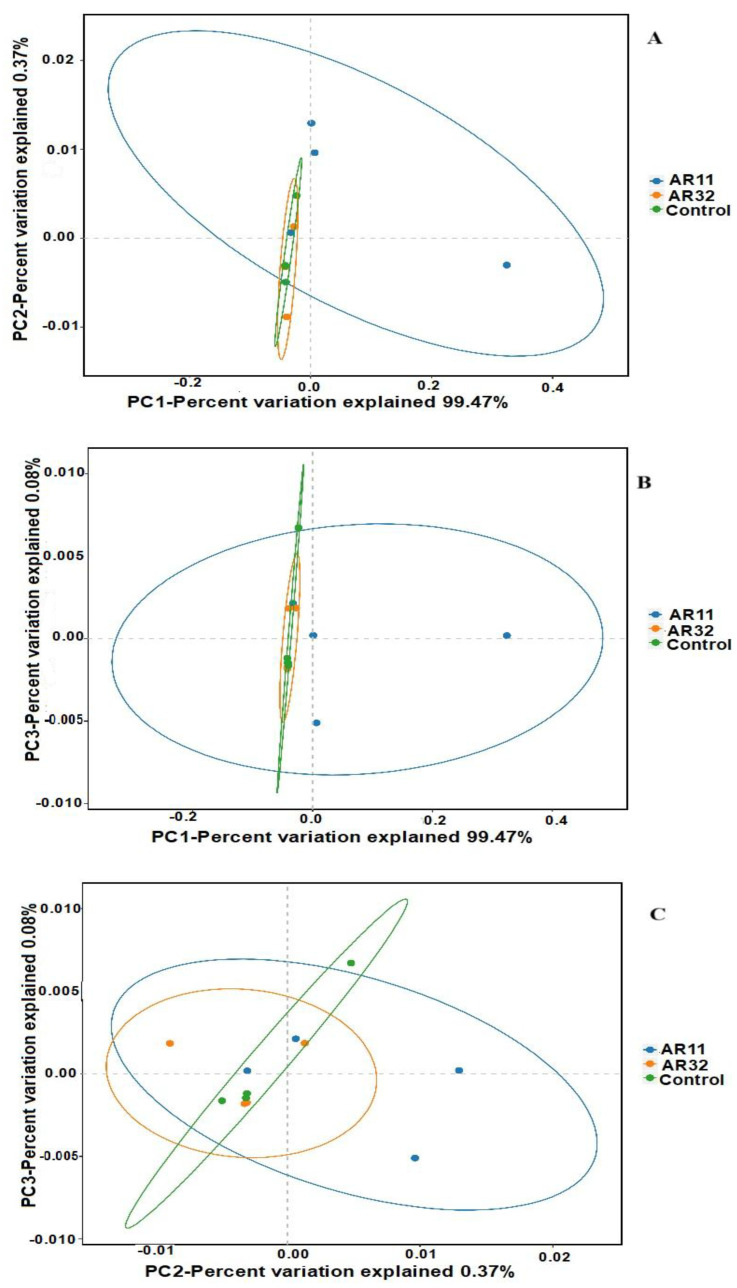
PCoA of beta diversity: weighted UniFrac-based PCoA plots ((**A**): PC1 vs. PC2, (**B**): PC1 vs. PC3, and (**C**): PC2 vs. PC3). AR11 and AR32: inoculated plant with two different bacteria strains.

Beta diversity was analyzed using principal coordinate analysis (PCoA) based on weighted UniFrac distances (Figure 5). The PCoA plots clearly illustrate differences in microbial community structures among the treatment groups (AR11, AR32, and control). Samples inoculated with AR11 formed distinct clusters in all comparisons (PC1 vs. PC2, PC1 vs. PC3, and PC2 vs. PC3), separating clearly from both the AR32 and control groups. This indicates significant shifts in microbial community composition and phylogenetic diversity due to AR11 inoculation. In contrast, AR32 samples showed partial overlap with the control group, suggesting more moderate changes in microbial community structure. The weighted UniFrac metric effectively captured the differences in microbial composition and phylogenetic structure, revealing that AR11 inoculation significantly altered the abundance and diversity of specific microbial taxa.

These findings highlight AR11’s potential to foster beneficial microbial communities in the rhizosphere, potentially improving plant health and resilience to stress. Additional analyses using non-metric multidimensional scaling (NMDS) and principal component analysis (PCA), presented in the Supplementary Materials (Figure S3), further support the clustering patterns observed in the PCoA analysis. These visualizations reinforce the consistent and significant impact of AR11 inoculation on microbial community composition across different analytical methods.

**Figure 6:**
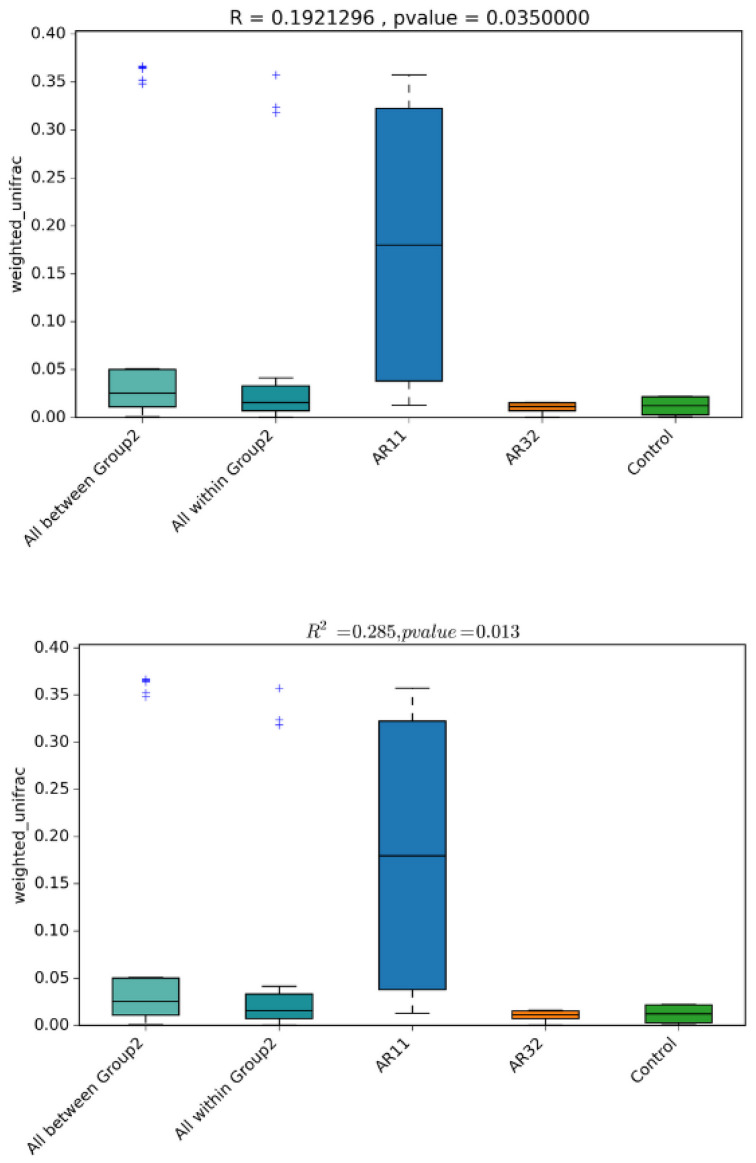
Boxplots of beta diversity metrics (weighted UniFrac) illustrate microbial community dissimilarity across the AR11, AR32, and control treatments. R-values from ANOSIM and R^2^-values from PERMANOVA quantify the strength of clustering among treatments, with *p*-values indicating statistical significance. AR11 and AR32: plant inoculated with two bacteria strains. The “+” symbols in the boxplot represent outliers, which are data points that differ significantly from the rest of the dataset.

Diversity analysis revealed significant shifts in microbial community composition between the *Bacillus* treated groups (AR11 and AR32) and the control. Weighted UniFrac distances indicate that both taxonomic identity and abundance contribute to these differences, with AR11 exhibiting the most pronounced changes (Figure 6). PERMANOVA (R^2^ = 0.285, *p* = 0.013) and ANOSIM (R = 0.192, *p* = 0.035) confirm significant group clustering, reinforcing AR11’s strong effect on microbial diversity. These findings suggest that *Bacillus* inoculation, particularly with AR11, leads to substantial restructuring of the pea plant microbiota.

### 3.4. Taxonimic Anotation

**Figure 7:**
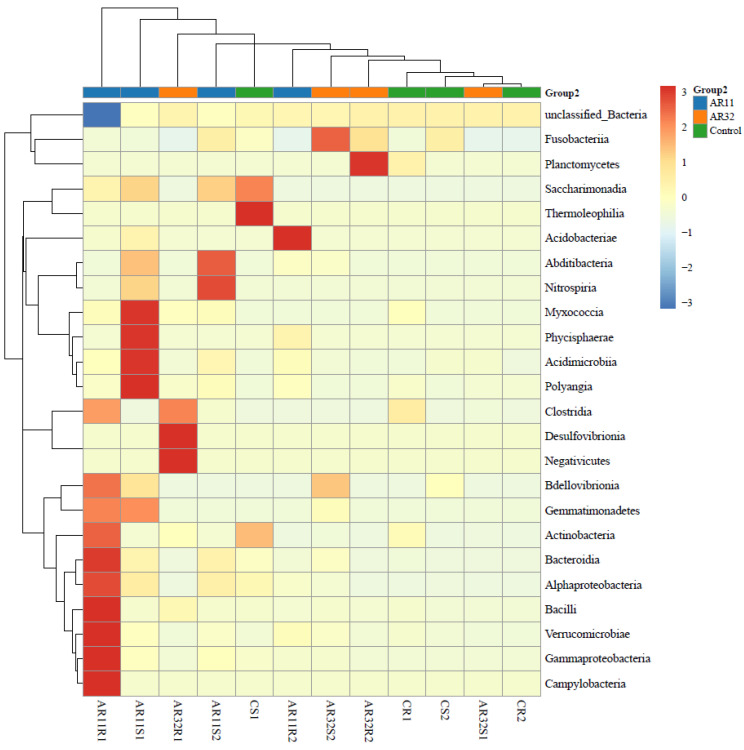
Taxonomic composition of bacterial communities in AR11, AR32, and control treatments. Heatmap showing the relative abundance of bacterial class across treatments. AR11 and AR32: plant inoculated with two bacterial strains.

Inoculation of pea seeds with *Bacillus* strains AR11 and AR32 resulted in significant changes in the bacterial community structure at the class level, as shown in the heatmap (Figure 7). Notable shifts were observed in the relative abundance of *Bacilli*, *Alphaproteobacteria*, *Gammaproteobacteria*, and *Bacteroidia*. Specifically, AR11-treated samples exhibited an enrichment of *Bacilli* and *Fusobacteria*, while AR32-treated samples showed an increased abundance of *Alphaproteobacteria* and *Acidobacteriae* compared to the control group. At the genus level, these changes were reflected in the increased abundance of *Bacillus*, *Pseudomonas*, *Rhizobium*, *Streptomyces*, and *Serratia* in AR11 and AR32 treatments (Supplementary Figure S4). More specifically, AR11 treatments were associated with a higher presence of *Serratia* and *Bacillus*, while AR32 treatments exhibited increased levels of *Pseudomonas* and *Rhizobium*.

**Figure 8:**
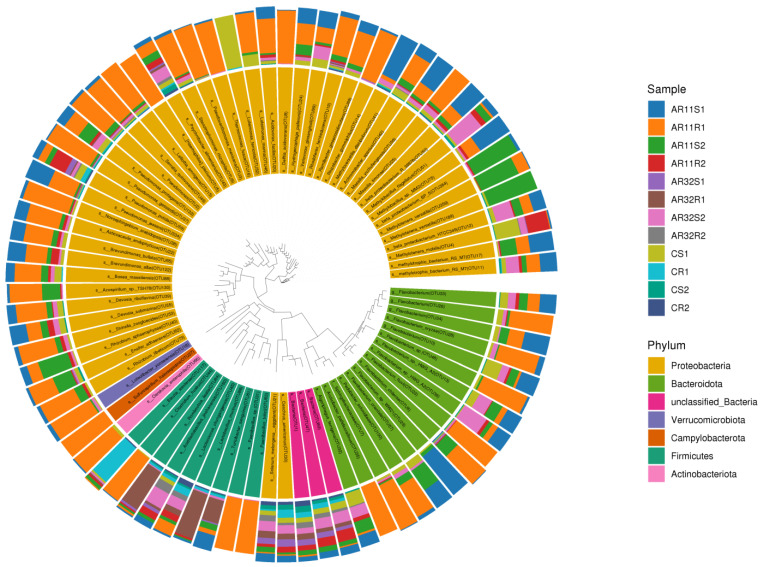
Phylogenetic tree displaying the taxonomic composition and evolutionary relationships of bacterial communities. The concentric rings represent taxonomic classifications from phylum to genus, with color-coded segments highlighting dominant taxa across. AR11: samples inoculated with bacterial strain AR11, AR32: samples inoculated with bacterial strain AR32, control (C): samples without bacterial inoculation. S: stem samples; R: root samples. 1, 2, 3: biological replicates.

The circular phylogenetic tree (Figure 8) provides a detailed visualization of the taxonomic distribution of bacterial communities across the samples. It highlights the relative abundance of various bacterial taxa at the phylum level, with *Proteobacteria*, *Firmicutes*, and *Actinobacteria* being clearly highlighted. Notably, samples treated with *Bacillus* strains AR11 and AR32 (AR11S1, AR11R1, AR32S1, AR32R1) exhibited distinct shifts in their microbial profiles compared to the control samples (CS1, CS2, CR1, CR2). These changes were characterized by an enrichment of beneficial taxa such as *Bacillus*, *Pseudomonas*, and *Rhizobium* in inoculated treatments, aligning with the observed promotion of plant growth in these groups. *Proteobacteria* remained dominant across all samples, but an increased proportion of *Firmicutes* and *Actinobacteriota* was observed in AR11and AR32 treated samples, reflecting the specific contribution of these strains in altering community composition.

### 3.5. Correlation Network of Microbial Communities

**Figure 9:**
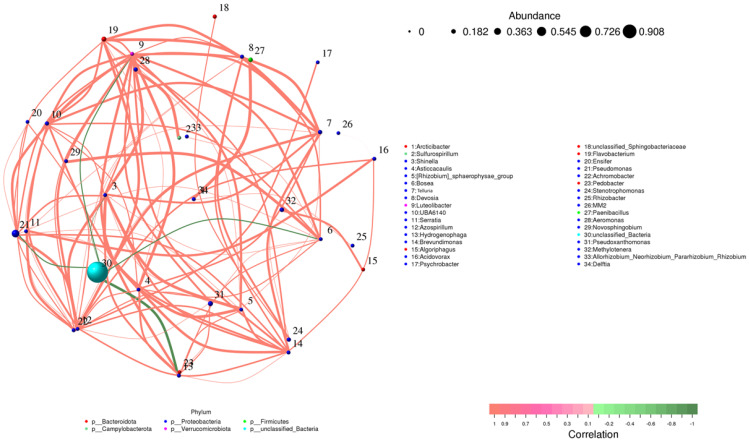
Correlation network of microbial genera across all samples (inoculated and control). Nodes represent bacterial genera, sized by relative abundance and colored by phylum. Edges denote significant correlations (Spearman correlation, *p* < 0.05, |r| > 0.6): red for positive correlations and green for negative correlations. Genera with weak or insignificant correlations do not appear in the network but are still listed in the legend based on classification.

The microbial community network consisted of thirty-four bacterial genera found in both the inoculated and control samples (Figure 9). In this network, each node represented a specific genus, with larger nodes indicating higher relative abundance. For example, *Pseudoxanthomonas* (Node 30) was the most abundant genus in the network. The network also revealed a higher number of positive correlations (represented by red edges), indicating cooperative interactions between different taxes. Genera from the phylum *Proteobacteria* formed tight clusters, underscoring their central role in the community. Notably, Pseudomonas (Node 21) and Delftia (Node 34) were strongly connected to several other genres, suggesting they play a crucial role in maintaining community stability and fostering cooperation among taxes. While negative correlations were less frequent, they were still notable, highlighting competitive interactions between certain genera. This balance between cooperation and competition illustrates the complexity of microbial communities. Inoculation with *Bacillus* strains further enhanced the abundance of beneficial genera, such as Rhizobium (Node 33) and Methylotenera (Node 32), which showed positive connections with key taxa. These genres are known for their roles in nitrogen fixation and promoting plant growth. This reinforces the idea that Bacillus inoculation boosts the presence of beneficial microbes in the rhizosphere, supporting plant health and growth.

### 3.6. Bacterial Community Composition at the Phylum Level Across Treatments

**Figure 10:**
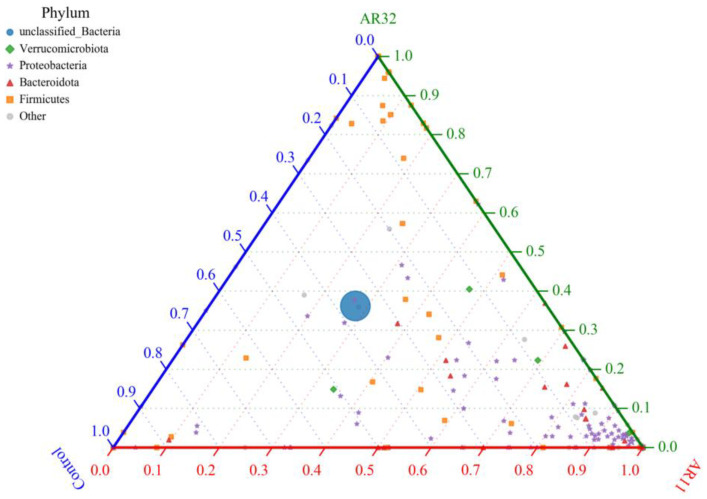
Ternary plot of bacterial phyla distribution across treatments nodes represents bacterial phyla, with sizes corresponding to relative abundance. Colors indicate different phyla. The axes represent AR11, AR32, and control treatments. AR11 and AR32: inoculated plant with two different bacteria strains.

The ternary plot illustrates the distribution of bacterial phyla among the AR11, AR32, and control treatments, highlighting shifts in microbial community composition (Figure 10). *Proteobacteria*, a phylum known for its plant growth promoting properties, was significantly enriched in AR11 treated plants, dominating the microbial community compared to AR32 and Control treatments. Meanwhile, *Firmicutes*, which are associated with biofilm formation and stress tolerance, showed a higher relative abundance in AR32 treated plants. In contrast, the control group had a higher proportion of unclassified bacteria, suggesting a less structured and potentially less beneficial microbial community. These findings indicate that while *Bacillus* species are commonly found in the plant microbiome, our study demonstrates that specific *Bacillus* inoculations (AR11 and AR32) can significantly alter the composition of the bacterial community. AR11 promoted the dominance of *Proteobacteria*, while AR32 favored Firmicutes. These changes play a role in delivering treatment specific benefits through enhanced plant microbe interactions.

### 3.7. Functional Profiles of Bacterial Communities Across Treatments

**Figure 11:**
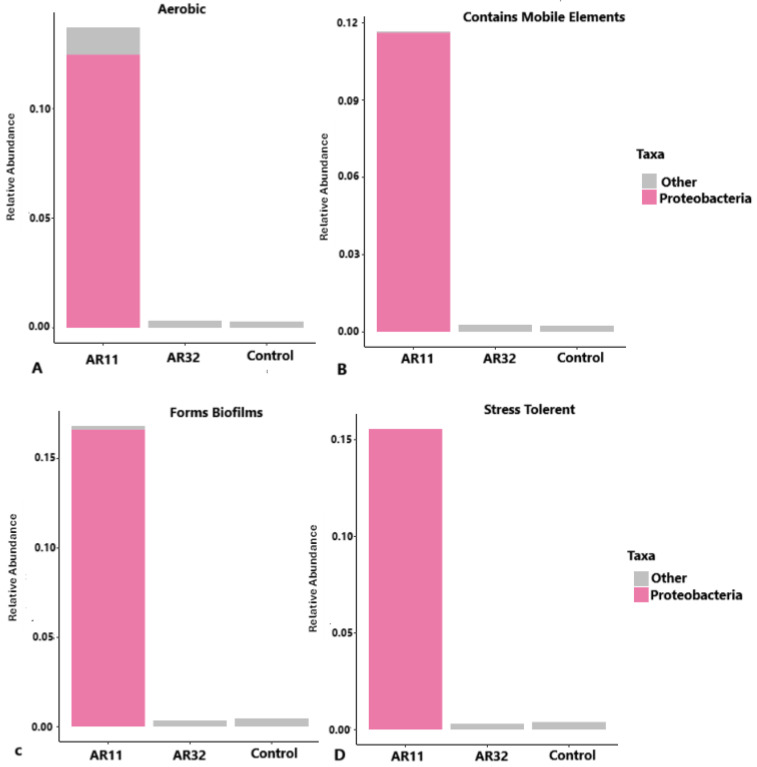
Functional profiles of bacterial communities across AR11, AR32, and control treatments. Bar plots represent the relative abundance of bacterial taxa categorized by key functional traits ((**A**) aerobic, (**B**) mobile genetic elements, (**C**) biofilm formation, (**D**) stress tolerance). Taxa are grouped into Proteobacteria and other phyla. AR11 and AR32: inoculated plant with two different bacteria strains.

The functional traits of bacterial communities in AR11, AR32, and control samples revealed distinct patterns of metabolic activity, genetic adaptability, and stress tolerance. AR11-treated samples exhibited a significantly higher abundance of aerobic bacteria, primarily from the phylum *Proteobacteria*, indicating enhanced nutrient cycling and plant growth (Figure 11). Mobile genetic elements, which facilitate horizontal gene transfer, were most prominent in AR11-treated samples, potentially enabling the spread of genes related to stress resistance and nutrient acquisition. Biofilm formation potential was also the highest in AR11-treated samples, highlighting their importance in root colonization and protection against environmental stress. Additionally, AR11 samples were enriched with stress tolerant bacteria, fostering a microbial community that enhances plant resilience under challenging conditions. Together, these findings demonstrate how *Bacillus* inoculation differentially shapes microbial communities to support plant health and growth.

## 4. Discussion

The findings of this study provide valuable insights into the effects of *Bacillus* inoculation on the microbial diversity and composition within pea plants. The observed variation in microbial richness, diversity, and community structure underscores the significant role of bacterial inoculants in modulating plant associated microbiomes. These effects are further influenced by the chemical properties of the soil, which indirectly support microbial diversity and activity. In this study, high organic carbon content, optimal nitrogen levels, and a slightly alkaline pH (6–8) created favorable conditions for microbial activity, aligning with optimal ranges for maximizing diversity [16,17,18]. Additionally, the high levels of macronutrients, such as potassium and phosphorus, likely enhanced soil fertility and strengthened plant microbe interactions, contributing to the establishment and performance of the introduced *Bacillus* strains [16,19,20]. Together, these factors fostered the development of enriched plant associated microbial communities, underscoring the combined role of soil properties and bacterial inoculants in driving microbial dynamics.

### 4.1. Enhancing Microbial Diversity with Bacillus Strains

The alpha diversity metrics (ACE, Chao1, Shannon, and PD Whole Tree indices) revealed that AR11 inoculation significantly increased microbial richness and evenness compared to AR32 and control treatments. This finding underscores AR11’s potential to facilitate the recruitment and proliferation of a more diverse microbial community, which is often associated with enhanced plant health, resilience to environmental stresses, and improved ecosystem functionality. In contrast, AR32 inoculation demonstrated a moderate effect, suggesting a less pronounced influence on microbial diversity. These results align with previous research demonstrate that microbial inoculants can effectively reshape plant-associated microbiomes, often in a context dependent manner [21,22]. The inoculation of seeds or early-stage plants with microbial strains has been shown to modulate microbial diversity and functionality, thereby facilitating plant adaptation to specific environmental conditions [23]. AR11 treated plants showed increased microbial richness, which can enhance nutrient cycling, pathogen suppression, and overall plant fitness, as reported in other studies on microbial inoculants [24,25,26].

The rarefaction and rank abundance curves further supported these findings. AR11 treated samples reached a plateau more rapidly and exhibited greater species evenness compared to controls, indicating a reduction in microbial dominance and a more balanced community structure. A balanced microbiome not only mitigates the risk of opportunistic pathogens but also improves plant tolerance to abiotic stresses, such as drought and salinity [27,28]. Moreover, microbial inoculants like AR11 can promote sustainable agriculture by reducing the need for chemical fertilizers and pesticides, thereby minimizing environmental harm [29,30]. The moderate effect of AR32, while still beneficial, highlights the specificity of plant microbe interactions and the need to identify strains best suited for particular crops or environmental conditions. This specificity is key to microbiome engineering, which leverages microbial inoculants to improve plant performance and stress tolerance [31]. Overall, the findings suggest that AR11 has significant potential as an efficient microbial inoculant for sustainable agricultural practices.

### 4.2. Shifts in Beta Diversity and Microbial Community Composition

Beta diversity analyses, including hierarchical clustering, NMDS, PCoA, and PCA, revealed distinct microbial community structures among treatments. AR11-treated samples consistently clustered away from the AR32 and control groups, highlighting AR11’s pronounced effect on microbial composition. The significant enrichment of beneficial taxa such as *Methylotenera*, *Paenibacillus*, and *Flavobacterium* in AR11-treated plants suggests a strong potential for enhancing plant health, resilience, and productivity. These genera are known for their roles in nitrogen fixation, phosphorus solubilization, and bioactive compound production, which collectively improve plant growth and pathogen suppression [32,33,34].

The moderate impact of AR32 suggests that different *Bacillus* strains interact uniquely with the plant microbiome. While both AR11 and AR32 inoculations promoted shifts in microbial community structure, AR11 showed a stronger capacity to recruit beneficial taxa, which led to a more pronounced differentiation from the control group. These findings align with research demonstrating that microbial inoculants can reshape host microbiomes in a context dependent manner [35]. Specifically, microbial inoculants can alter plant microbiomes to enhance plant performance, resilience to stress, and overall productivity, as shown in several studies on plant microbe interactions [36].

AR32 treated plants exhibited a higher relative abundance of generalist taxa, which may contribute to baseline ecological functionality but lack the specialized interactions seen with AR11. This suggests that AR32 may promote a more general microbial community without fostering the specific beneficial interactions essential for enhanced plant growth. This is consistent with findings in other studies where specific microbial communities, rather than generalized ones, are responsible for significant plant growth promotion and stress tolerance [37,38].

The core microbiome analysis revealed a stable set of microbial taxa shared across all treatments, essential for maintaining plant health and basic physiological functions. However, AR11 inoculation expanded this core by introducing additional beneficial taxa, demonstrating its potential to augment the microbiome beyond the baseline established by control treatments. These findings suggest that specific microbial inoculants can expand the microbial diversity and functionality of plant associated microbiomes, enhancing the plant’s ability to adapt to environmental challenges.

### 4.3. Functional Contributions of the Microbial Community

The observed changes in microbial diversity and composition have functional implications for plant growth and health. Higher alpha diversity is associated with greater functional redundancy and ecosystem stability, which can improve the plant’s resilience to biotic and abiotic stresses. The enriched taxa in AR11-treated plants, such as *Paenibacillus* and Flavobacterium, are known to produce secondary metabolites with antimicrobial properties, which may protect plants against soilborne pathogens. Additionally, *Methylotenera* has been linked to efficient carbon metabolism and nutrient cycling, which could enhance the plant’s nutrient acquisition capabilities.

### 4.4. Practical Applications and Future Research

The findings of this study emphasize the potential of *Bacillus* inoculants, particularly AR11, as biostimulants for sustainable agriculture. By promoting microbial diversity and enriching beneficial taxa, AR11 inoculation can enhance plant health and productivity while potentially reducing the need for chemical fertilizers and pesticides. Future research should focus on the mechanistic understanding of plant microbe interactions facilitated by AR11, including the identification of signaling molecules and metabolic pathways involved. Field trials are essential to validate these results across different environmental conditions and cropping systems. The potential risk of competition with native microbes should also be considered, as it could affect the inoculant’s efficacy. Furthermore, meta transcriptomic and metabolomic analyses would offer valuable insights into the functional roles of microbial taxa enriched in AR11-treated plants. Interpreting microbiome shifts requires consideration of potential influencing factors, including soil composition, environmental conditions, and plant genotype variations. Soil properties such as nutrient availability, texture, and organic matter, along with environmental factors like temperature and humidity, can significantly influence microbial community dynamics [39]. Additionally, plant genotype differences may impact microbial colonization patterns [40], Future studies should account for these variables to achieve a more comprehensive understanding of microbial dynamics in natural and field conditions.

## 5. Conclusions

This study highlights the potential of *Bacillus* inoculants, particularly AR11, to modulate microbial diversity and composition within pea plants. The enrichment of beneficial taxa, alongside the enhancement of microbial evenness and diversity, suggests that AR11 inoculation can significantly contribute to plant health, resilience, and productivity. Notably, this is a novel molecular study of plant inoculation with non host bacteria, demonstrating the capacity to modulate the microbiome and introduce beneficial traits that can positively affect the plant. While this research provides valuable insights under controlled conditions, future studies conducted in field and real-world agricultural environments could further validate these findings. This work paves the way for ongoing research into microbial inoculants for sustainable agriculture, emphasizing their role in enhancing plant–microbe interactions and promoting ecological balance.

## Data Availability

Raw amplicon sequences will be available in the NCBI Sequence Read Archive (SRA) under the PRJNA1221229 at https://www.ncbi.nlm.nih.gov/sra/PRJNA1221229.

## Supplementary Materials

The following supporting information can be downloaded at: https://www.mdpi.com/article/10.3390/microorganisms13030570/s1, Figure S1: Beta diversity analysis of microbial community composition across treatments. (A) Binary Jaccard, (B) Bray–Curtis, and (C) unweighted UniFrac metrics were used to evaluate differences between the control group and groups inoculated with *Bacillus* strains AR11 and AR32; Figure S2: Beta diversity and taxonomic shifts induced by microbial inoculants. (A) Binary Jaccard, (B) Bray–Curtis, (C) unweighted UniFrac metrics, and (D) weighted UniFrac metric were used to evaluate differences between the control group and groups inoculated with *Bacillus* strains AR11 and AR32; Figure S3: Principal Component Analysis (PCA) of microbial community composition among treatment groups (AR11, AR32, and Control). (A) PCA plot of PC1 vs. PC2, (B) PCA plot of PC1 vs. PC3, and (C) PCA plot of PC2 vs. PC3. Each point represents a sample, and ellipses indicate 95% confidence intervals for each group; Figure S4: Taxonomic composition of bacterial communities in AR11, AR32, and control treatments. Heatmap shows the relative abundance of bacterial genus across treatments. AR11 and AR32: plants inoculated with two bacterial strains.
