# Supplementary material for: Modulating the Plant Microbiome: Effects of Seed Inoculation with Endophytic Bacteria on Microbial Diversity and Growth Enhancement in Pea Plants
Source: Microorganisms. 2025 Mar 3;13(3):570. doi: 10.3390/microorganisms13030570 (PMC11945133; doi:10.3390/microorganisms13030570)
Supplement: Supplementary file 1 [file microorganisms-13-00570-s001.zip › microorganisms-3476554-supplementary.pdf]

Supporting information

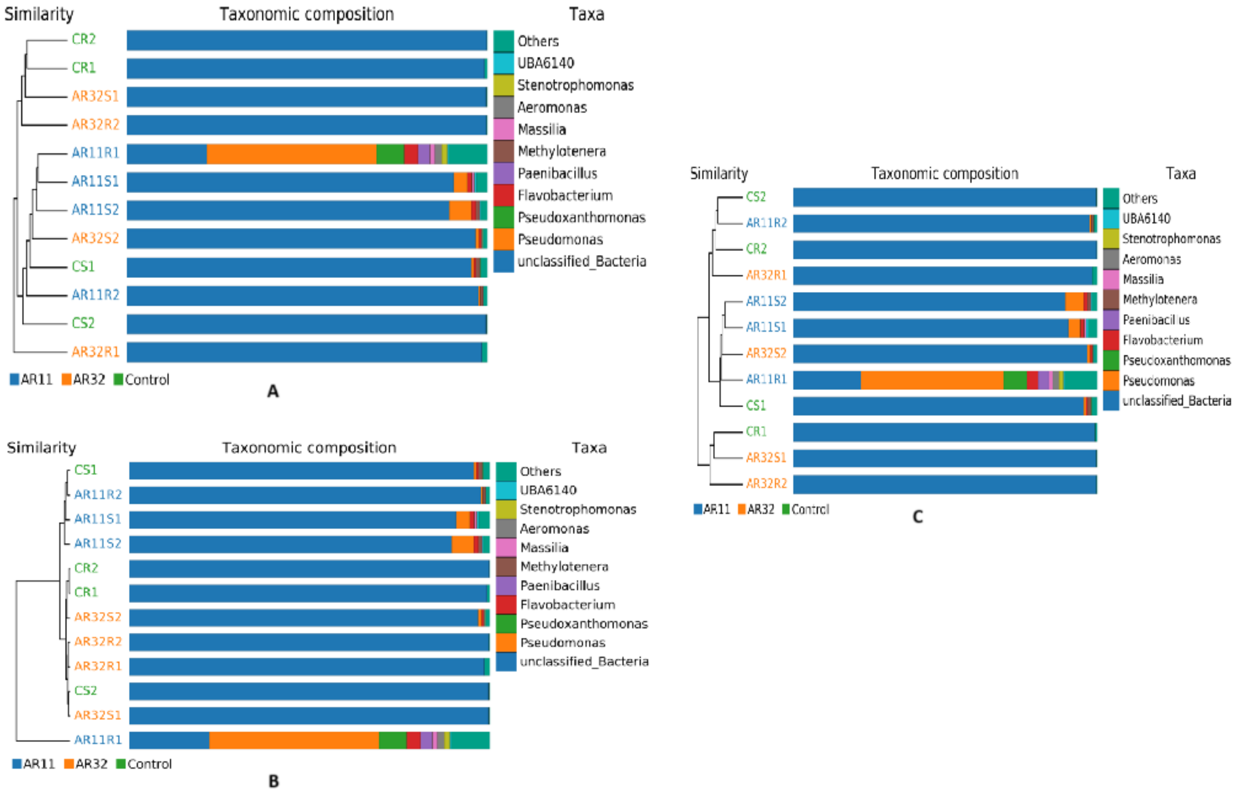

Figure S1. Beta diversity analysis of microbial community composition across treatments. (A) Binary Jaccard, (B) Bray-Curtis, and (C) Unweighted UniFrac metrics were used to evaluate differences between the Control group and groups inoculated with *Bacillus* strains AR11 and AR32.

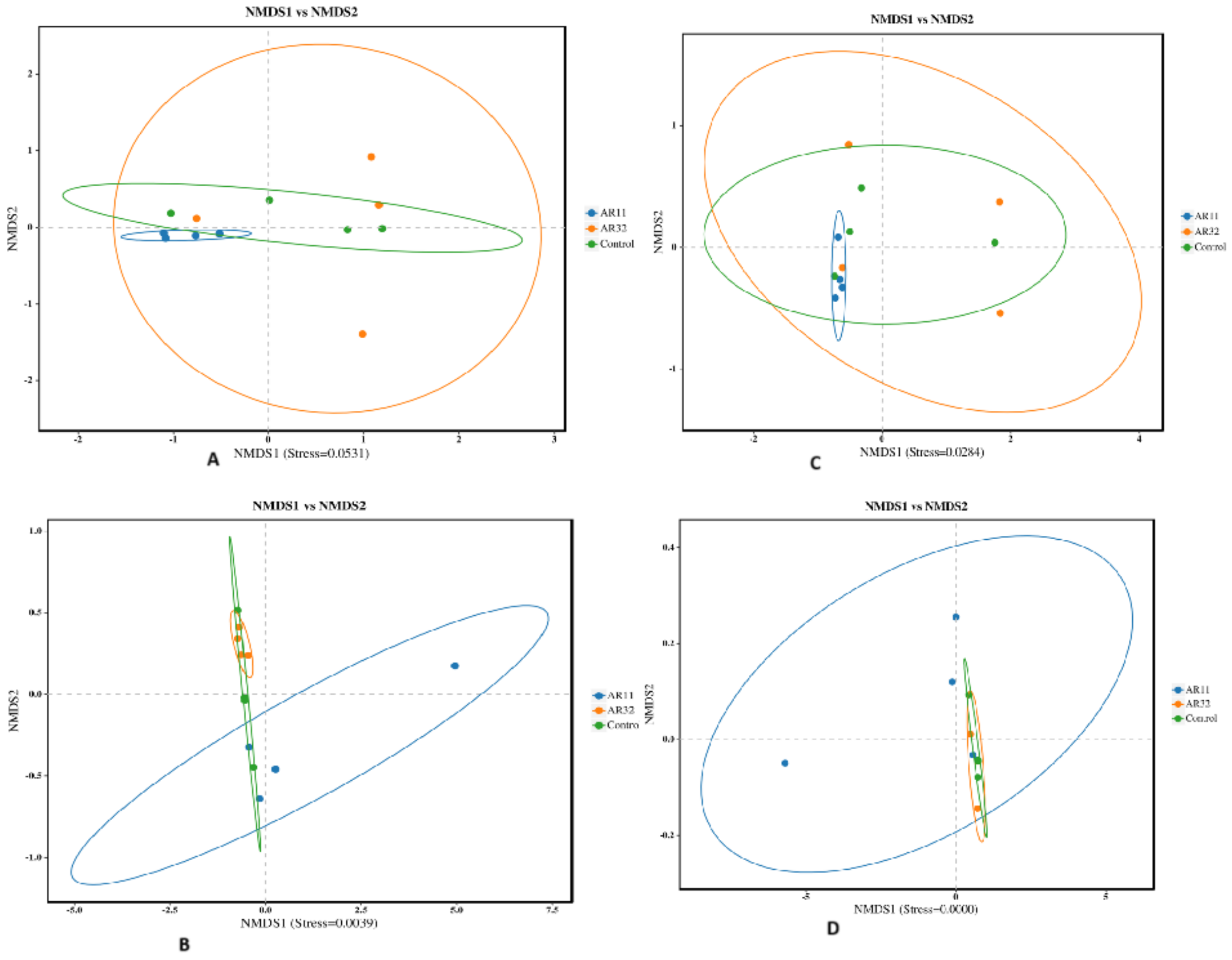

Figure S2. Beta Diversity and Taxonomic Shifts Induced by Microbial Inoculants. (A) Binary Jaccard, (B) Bray Curtis, (C) Unweighted UniFrac metrics, and (D) weighted UniFrac metric were used to evaluate differences between the Control group and groups inoculated with *Bacillus* strains AR11 and AR32.

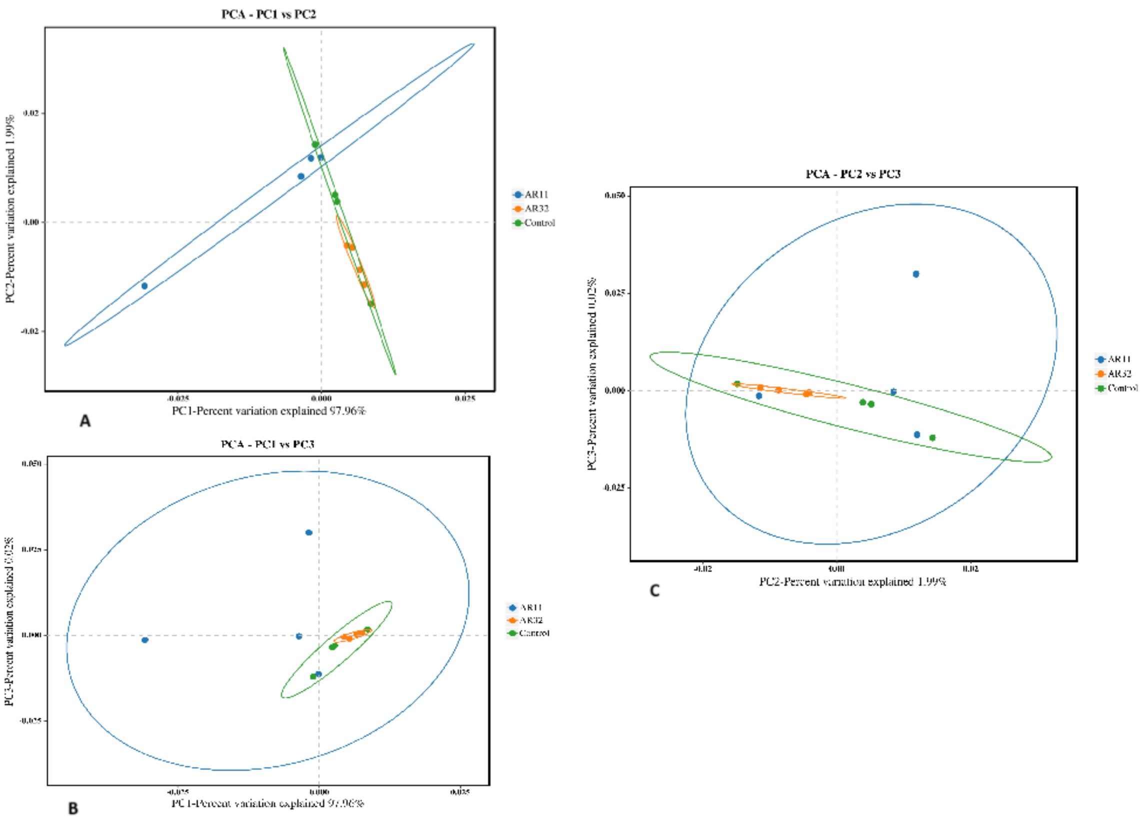

Figure S3. Principal Component Analysis (PCA) of microbial community composition among treatment groups (AR11, AR32, and Control). (A) PCA plot of PC1 vs. PC2, (B) PCA plot of PC1 vs. PC3, and (C) PCA plot of PC2 vs. PC3. Each point represents a sample, and ellipses indicate 95% confidence intervals for each group.

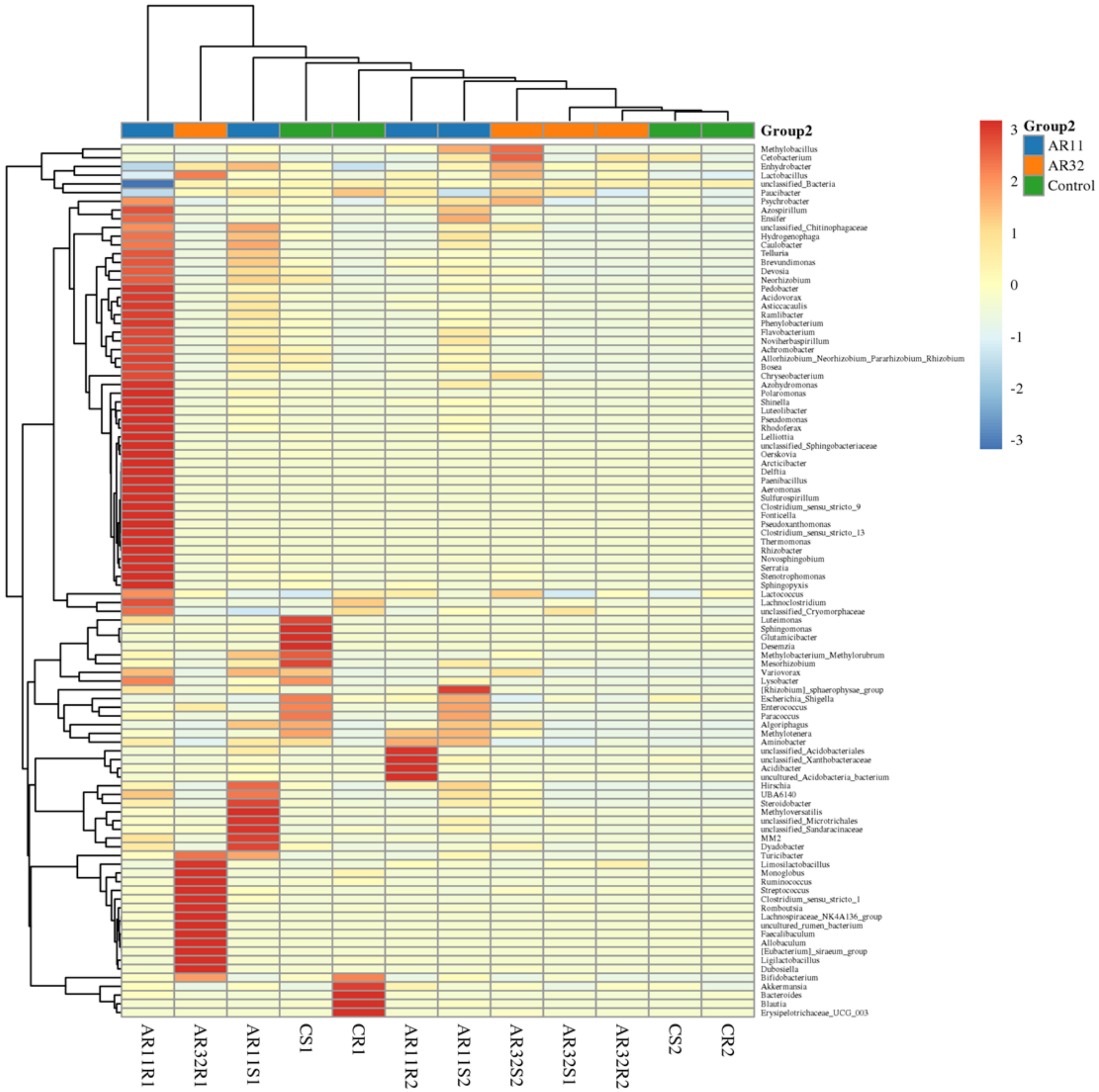

Figure S4. Taxonomic composition of bacterial communities in AR11, AR32, and Control treatments. Heatmap shows the relative abundance of bacterial genus across treatments. AR11 and AR32: Plants inoculated with two bacterial strains.
